# Extracting Clinical Features From Dictated Ambulatory Consult Notes Using a Commercially Available Natural Language Processing Tool: Pilot, Retrospective, Cross-Sectional Validation Study

**DOI:** 10.2196/12575

**Published:** 2019-11-01

**Authors:** Jeremy Petch, Jane Batt, Joshua Murray, Muhammad Mamdani

**Affiliations:** 1 Institute of Health Policy, Management and Evaluation Dalla Lana School of Public Health University of Toronto Toronto, ON Canada; 2 Centre for Data Science and Digital Health Hamilton Health Sciences Hamilton, ON Canada; 3 Division of Respirology Department of Medicine University of Toronto Toronto, ON Canada; 4 Keenan Research Centre for Biomedical Science St. Michael's Hospital Toronto, ON Canada; 5 Department of Medicine St. Michael's Hospital Toronto, ON Canada; 6 Li Ka Shing Centre for Healthcare Analytics Research and Training St. Michael's Hospital Toronto, ON Canada; 7 Department of Statistical Sciences Faculty of Arts and Sciences University of Toronto Toronto, ON Canada; 8 Leslie Dan Faculty of Pharmacy University of Toronto Toronto, ON Canada; 9 Department of Medicine Faculty of Medicine University of Toronto Toronto, ON Canada

**Keywords:** natural language processing, electronic health record, tuberculosis

## Abstract

**Background:**

The increasing adoption of electronic health records (EHRs) in clinical practice holds the promise of improving care and advancing research by serving as a rich source of data, but most EHRs allow clinicians to enter data in a text format without much structure. Natural language processing (NLP) may reduce reliance on manual abstraction of these text data by extracting clinical features directly from unstructured clinical digital text data and converting them into structured data.

**Objective:**

This study aimed to assess the performance of a commercially available NLP tool for extracting clinical features from free-text consult notes.

**Methods:**

We conducted a pilot, retrospective, cross-sectional study of the accuracy of NLP from dictated consult notes from our tuberculosis clinic with manual chart abstraction as the reference standard. Consult notes for 130 patients were extracted and processed using NLP. We extracted 15 clinical features from these consult notes and grouped them a priori into categories of simple, moderate, and complex for analysis.

**Results:**

For the primary outcome of overall accuracy, NLP performed best for features classified as simple, achieving an overall accuracy of 96% (95% CI 94.3-97.6). Performance was slightly lower for features of moderate clinical and linguistic complexity at 93% (95% CI 91.1-94.4), and lowest for complex features at 91% (95% CI 87.3-93.1).

**Conclusions:**

The findings of this study support the use of NLP for extracting clinical features from dictated consult notes in the setting of a tuberculosis clinic. Further research is needed to fully establish the validity of NLP for this and other purposes.

## Introduction

### Background

In recent years, the use of electronic health records (EHRs) in office-based clinical practices in the United States has more than doubled, from approximately 40% in 2008 to nearly 90% in 2015 [1]. This rise has been even sharper in hospitals, where EHR adoption has increased from about 10% in 2008 to nearly 85% in 2015 [2]. The increasing adoption of EHRs in clinical practice holds the promise of improving care and advancing research by serving as a rich source of data. However, gleaning useful information from EHR data can be challenging, and the use of such data for research purposes varies considerably across jurisdictions [3].

One challenge relates to EHRs allowing clinicians to enter data in text format without much structure. Although this enhances clinical usability, it often requires costly and time-consuming manual chart abstraction processes to extract useful information in a structured manner. These challenges have sparked an increasing interest in the potential for natural language processing (NLP) approaches to process unstructured clinical digital text data, extract clinical features, and convert them into structured data.

Although NLP approaches for processing radiological reports are now well established [4], the practice of using NLP for processing more general clinical documentation, especially consult notes, is still developing. Research to date has explored several applications of NLP to general clinical documentation, including identification of breast cancer recurrence [5], social isolation [6], falls risk [7], depression [8], homelessness [9], intraductal papillary mucinous neoplasms [10], and new clinically relevant information for organ transplant patients [11]. One common feature of much of the research to date is that studies have tended to leverage open-source and academic tools for NLP. Although these tools can be highly effective, most are available as libraries for programing languages such as Python and R, which can pose a barrier for health care organizations that lack robust digital capacity or academic partnerships. However, there are an increasing number of commercially available NLP tools, such as Linguimatics I2E and Google Cloud’s AutoML, that promise to make NLP significantly more accessible for general users, but to date, there have been relatively fewer studies that have evaluated the validity of these tools for clinical feature extraction [6,7,12].

### Objective

We conducted a pilot study to examine the accuracy of a commercially available NLP tool relative to manual chart abstraction in capturing useful information from free-text consult notes in an outpatient tuberculosis (TB) clinic.

## Methods

### Study Setting

We conducted a pilot, retrospective, cross-sectional study of feature extraction accuracy using NLP, with manual chart abstraction as the reference standard. The study setting was St. Michael’s Hospital, which is a 450-bed urban academic hospital affiliated with the University of Toronto. The St. Michael’s TB program serves as a tertiary referral center for patients with active TB and latent TB infection, managing patients in both inpatient and outpatient settings. The program is staffed by a rotating roster of 8 physicians (6 respirologists and 2 infectious disease physicians) and 1 TB nurse practitioner and has a volume of approximately 2000 outpatient encounters annually. This study was approved by the St. Michael’s Hospital Research Ethics Board and conducted in accordance with its policies.

### Natural Language Processing Approach

**Figure 1:**
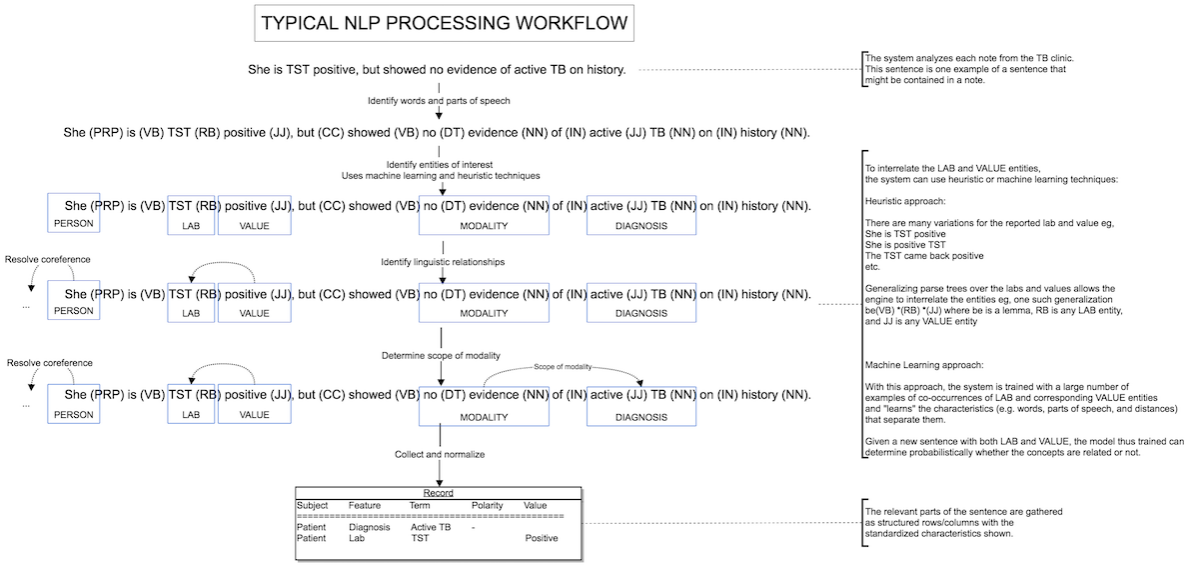
Natural language processing (NLP) workflow using the DARWEN tool. PRP: pronoun; VB: verb; RB: adverb; JJ: adjective; CC: coordinating conjunction; DT: determiner; NN: noun; IN: preposition; TB: tuberculosis; TST: Tuberculin Skin Test.

We conducted our NLP analysis using a commercial NLP engine (Pentavere’s DARWEN), which integrates 3 primary approaches to extract clinical features: (1) manually prepared natural language extraction rules that describe the general syntax and lexicon of each feature (both custom and internationally recognized ontologies such as Medical Subject Headings and Systematized Nomenclature of Medicine-Clinical Terms are utilized as an initial source of synonyms for common clinical terms), (2) machine-learned inferred rules that are designed to complement and reduce the extraction error rate of the manually prepared rules (the usage of machine learning in DARWEN is directed to improve the quality of the clinical natural language extraction rather than to predict or infer clinical features based on other features, as is the case with many competing systems), and (3) heuristic rules that encapsulate overarching clinical knowledge that must be respected when considering the clinical features holistically. This workflow is illustrated in Figure 1.

We followed the standard process for employing DARWEN, which involves tuning, testing, and retuning against a reference standard, together with clinician consultation to resolve any semantic issues as well as to develop the heuristic rules. Tuning refers to the process of refining NLP extraction rules based on manual analysis of text and is an essential step to successfully account for the variability in terminology and documentation structure between clinicians. Generating rules during the tuning process is an iterative, feature-by-feature, semisupervised process. First, we focused on recognizing the key entities associated with any feature, such as comorbidities. Given the low volume of data in the training set, we started with recurrent neural network-based named entity recognition (NER) models, which were pretrained for recognizing drugs, diagnosis, medical risk factors, and adverse drug reactions on Pentavere’s proprietary clinical dataset (Pentavere’s proprietary corpus includes over 100,000 patients, with an average of 50 clinical notes per patient); discussed the match results with the clinician; and supplemented the NER model with heuristics to accommodate any discrepancies. For clinical features not appropriate for NER models, we employed a purely heuristic approach. For example, for a feature such as smoking status, we developed an initial set of rules to cover 3 straightforward cases: explicit mention of nonsmoker (eg, “She never smokes”), explicit mention of former smoker (eg, “she is a former light smoker”), and qualified mention of former smoker (eg, “She is a smoker who gave up 2 years ago”). Although these captured many cases of smoking found in the text, the tuning process revealed many more subtle cases that require further development of rules, such as a smoker who quit and then started again, handling of indeterminant language (eg, “She has a 20 pack year smoking history” in which it is not clear whether the patient still smokes or has quit), oblique mentions (eg, “She uses marijuana”), and second-hand smoker (eg, “Her former roommate was a smoker, but she was not.”) In this case, we developed rules to label token sequences (spans) into each of the different cases of smoker, former smoker, and nonsmoker. These rules are a combination of syntactic and lexical patterns, sometimes manually specified and sometimes induced from the data itself.

We then turned our attention to modeling the relationships between entities using a constituent parse tree kernel–based induction semisupervised machine learning technique, Pentavere’s proprietary algorithm inspired by the Dual Iterative Pattern Relation Expansion algorithm [13]. For training data, the algorithm uses a few starting phrases or sentences that provide a valid relationship and a few that provide an invalid relationship. Given some initial examples of related entities, the algorithm finds generalizations of parse trees that define those known relationships. These syntactic rules/patterns were then applied to find other entities that appear to be in similar relationships. We also leveraged features of the tool that support several contextual states, including polarity (negation), certainty/uncertainty, hypothesis (if… then…), historical context (history of…), and experiencer (patient and family member). This contextualization uses constituent and dependency parse trees to describe different types of relationships between tokens in text and thus determine the scope of the context, for example, to restrict a context to only apply to entities contained in specific sub (constituent) trees of the context and/or require a specific dependency relationship between the entities in context. For a case such as, “She has no apparent rash causing her pruritus,” this approach recognizes that rash is negated but pruritus is not negated.

### Sampling Approach

To create our corpus, we randomly sampled 130 patient records from a total pool of 351 records from our hospital’s outpatient TB clinic without exclusion and extracted their consult notes from their EHR. Consult notes for all outpatient encounters in the TB clinic are dictated by the attending physician or resident, followed by review and electronic sign-off by the attending physician. Dictations are free format, with no standardized template. They contain detailed clinical information about patients’ demographics, diagnosis, treatment course (including medications), and progress. Given that these notes contain personal health information, we are not able to share the corpus, but we have included synthetic samples of both assessment and follow-up notes in Multimedia Appendix 1, which are representative of the corpus.

We randomly divided our sample into 3 parts to support the tuning process described above, a tuning sample (n=30), a first-round testing/retuning sample (n=50), and a final testing sample (n=50). A single patient record allotted to the final testing sample contained corrupted data, reducing the final testing sample size to 49.

### Feature Identification

The following features were selected for extraction: country of birth, date of immigration to Canada, HIV status, known TB exposure, previous TB, smoking status, diagnosis, method of diagnosis, TB sensitivities, sputum culture conversion date, drug treatments, adverse drug reactions, medical risk factors for TB acquisition, social risk factors for TB acquisition, and disease extent (Table 1).

**Table 1:** Feature categorization based on a priori assessment of clinical and linguistic complexity.

Feature complexity and feature		Type	Examples
**Simple**			
	Country of birth	Country	India; Indonesia
	Date of immigration	Date	30/06/2013
	Smoking status	Categorical	Current smoker; former smoker
	Drug treatment	Text mapped to drug list	Isoniazid; rifampin
**Moderate**			
	HIV status	Binary	Positive/negative
	Known TB^a^ exposure	Binary	Yes/no
	Previous TB	Binary	Yes/no
	Method of diagnosis	Categorical	Culture positive; polymerase chain reaction positive
	TB sensitivities	Categorical	Fully sensitive; isoniazid resistant
**Complex**			
	Diagnosis	Categorical	Active TB; latent TB infection
	Sputum conversion date	Date	22/07/2016
	Adverse drug reactions	Categorical	Peripheral neuropathy; rash
	Medical risk factors	Categorical	Chemotherapy; renal failure
	Social risk factors	Categorical	Refugee camp resident; jail inmate
	Disease extent	Categorical	Pulmonary acid fast bacilli smear positive; disseminated

^a^TB: tuberculosis.

For each feature where a patient could have multiple observations, a series of dichotomous indicator features were created. For example, for drug treatment, patients could be on multiple medications, so dichotomous features were created for each relevant medication.

For analysis, we pooled these features into 3 categories—simple, moderate, and complex—based on an a priori assessment by a clinical expert of the relative clinical and linguistic complexity of each feature, based upon their clinical judgment (Table 1). Complex features were typically those where NLP would have to go well beyond simply categorizing terms based on a reference dictionary but would instead have to successfully process rich language with significant clinical context. For example, adverse drug reactions are particularly challenging as we may see the mention of a *rash* in the text, but this does not determine whether there was in fact a rash or whether a rash was the result of an adverse drug reaction. To determine whether there was a rash, we have to be able to rule out cases with the physician dictating “no evidence of rash,” patient complaining of rash but not diagnosed as such by the physician, and the physician dictating that she discussed rashes as possible side effects of the medication. Once it has been determined that a rash is present, we must first determine whether a rash is in fact a possible side effect of a drug the patient had been prescribed and then identify if the rash started when the drug was administered, which unless explicitly dictated, requires the solution to process the patient encounters longitudinally.

The reference standard was created by manually extracting features from patient records using a standardized data extraction form by a trained chart reviewer to serve as the *reference standard analysis*. One of the coauthors (JB) trained both the chart reviewer and the NLP engineer on how to perform chart abstraction to ensure the same clinical criteria would be used by both. This coauthor (JB) performed arbitration in cases of disagreement between the chart abstractor and the NLP tool’s output. Arbitrated results were used to retune the model on the training dataset before the final testing phase.

### Statistical Analysis

The primary outcome of our study was overall accuracy, defined as the number of correctly classified observations divided by the total number of observations [14]. Secondary outcomes were sensitivity (recall), specificity, positive predictive value (PPV; precision), and negative predictive value (NPV) [15,16]. NLP-abstracted data were treated as the *index analysis*, with manual chart review acting as the *reference standard analysis*.

Analysis was divided into 2 stages. The first stage was conducted after a single round of tuning of the NLP algorithms (n=50). The results of this stage were used to retune the semantic and heuristic rules used by the NLP tool to improve accuracy. The final analysis stage was conducted on the remaining records (n=49).

For the primary outcome, within each feature category, we calculated the accuracy and a 95% CI using standard methods for continuous features and proportions [17]. For secondary outcomes, we calculated the average and standard deviation within each category. For example, for the simple category, we calculated secondary outcomes for each feature within the category, averaged them, and calculated the standard deviation. This is a way of illustrating the average sensitivity, specificity, PPV and NPV, and spread across all classes of a multicategorical feature. All analyses were conducted using R (v 3.3.0).

## Results

### Overview

The study sample of 129 subjects included 71 females (55.0%, 71/129) with a mean age of 36.51 years and 58 males (45%) with a mean age of 46.74 years. Consult notes from 9 clinicians (8 physicians and 1 nurse practitioner) were included in the sample. A total of 138 points of discrepancy between the NLP process and the reference standard chart abstraction were identified.

### Natural Language Processing Performance

For the primary outcome (Table 2), NLP performed best for features classified as simple, achieving an overall accuracy of 96% (95% CI 94.3-97.6). Performance was slightly lower for features of moderate clinical and linguistic complexity at 93% (95% CI 91.1-94.4) and lowest for complex features at 91% (95% CI 87.3-93.1).

For secondary outcomes (Table 2), NLP achieved a sensitivity of 94% (SD 7.7) for simple, 60% (SD 38.6) for moderate, and 74% (SD 45.7) for complex features and PPV of 96% (SD 6.4) for simple, 70% (SD 33.7) for moderate, and 54% (SD 37.4) for complex features. The relatively low sensitivity and PPV for moderate and complex features is in contrast to its specificity of 99% (SD 0.5) for simple, 94% (SD 5.0) for moderate, and 89% (SD 8.3) for complex features and NPV of 99% (SD 1.7) for simple, 96% (SD 6.6) for moderate, and 98% (SD 2.9) for complex features.

Unsurprisingly, we saw considerable variation in NLP’s performance at the clinical feature level (Table 3). NLP performed extremely well for detecting drug prescriptions, achieving 100% for all primary and secondary outcomes for moxifloxacin, rifampin, ethambutol, and isoniazid. In contrast, NLP did not perform well at the feature level when measuring disease extent, with a sensitivity of only 25% for pulmonary acid fast bacilli (AFB) positive and 0% for extra pulmonary cases because of a very low number of these cases in our sample (4 pulmonary AFB-positive cases and 2 extrapulmonary cases).

**Table 2:** Primary and secondary outcomes for natural language processing (index analysis) compared with manual chart review (reference standard analysis).

Feature complexity	Primary outcome, overall accuracy (95% CI)	Secondary outcomes			
		Sensitivity/recall (SD)	Specificity (SD)	Positive predictive value/precision (SD)	Negative predictive value (SD)
Simple	96.3 (94.3-97.6)	93.8 (7.7)	99.7 (0.5)	96.4 (6.4)	99.0 (1.7)
Moderate	92.9 (91.1-94.4)	60.2 (38.6)	94.2 (5.0)	70.2 (33.7)	95.6 (6.6)
Complex	90.6 (87.3-93.1)	73.8 (45.7)	89.2 (8.3)	53.6 (37.4)	98.4 (2.9)

**Table 3:** Primary and secondary outcomes for natural language processing (index analysis) compared with manual chart review (reference standard analysis) at the clinical feature level.

Feature			Primary outcome, overall accuracy (95% CI)	Secondary outcomes^a^			
				Sensitivity/recall (SD)	Specificity (SD)	Positive predictive value/precision (SD)	Negative predictive value (SD)
**Simple features**							
	Country of birth		0.92 (0.80-0.98)	0.88 (0.32)	0.99 (0.01)	0.97 (0.11)	0.99 (0.01)
	Year of immigration		0.90 (0.78-0.97)	0.89 (0.29)	0.99 (0.02)	0.98 (0.08)	0.99 (0.01)
	Smoking status		0.94 (0.83-0.99)	0.92 (0.08)	0.98 (0.03)	0.85 (0.30)	0.97 (0.02)
	Sputum conversion date		0.98 (0.89-0.99)	0.80 (0.45)	0.99 (0.01)	0.99 (0.01)	0.99 (0.01)
	Pyrazinamide		0.96 (0.86-0.99)	1.00	0.85	0.95	1.00
	Moxifloxacin		1.00 (0.93-1.00)	1.00	1.00	1.00	1.00
	Vitamin B6		0.92 (0.80-0.98)	1.00	0.86	0.84	1.00
	Rifampin		1.00 (0.93-1.00)	1.00	1.00	1.00	1.00
	Ethambutol		1.00 (0.93-1.00)	1.00	1.00	1.00	1.00
	Isoniazid		1.00 (0.93-1.00)	1.00	1.00	1.00	1.00
	Levofloxacin		0.98 (0.89-0.99)	N/A^b^	0.98	N/A	N/A
**Moderate features**							
	HIV status		0.94 (0.83-0.99)	0.94	0.94	0.89	0.97
	TB^c^ contact		0.82 (0.68-0.91)	0.80	0.82	0.67	0.90
	Old TB		0.94 (0.83-0.99)	0.71	0.98	0.83	0.95
	Culture positive		0.88 (0.75-0.95)	0.33	1.00	1.00	0.87
	Polymerase chain reaction positive		1.00 (0.93-1.00)	1.00	1.00	1.00	1.00
	Clinical diagnosis		1.00 (0.93-1.00)	1.00	1.00	1.00	1.00
	Drug sensitivity		0.92 (0.80-0.98)	0.81 (0.27)	0.97 (0.04)	0.73 (0.25)	0.91 (0.14)
	Corticosteroids		0.98 (0.89-0.99)	N/A	0.98	N/A	N/A
	Chemotherapy		0.94 (0.83-0.99)	0.50	0.96	0.33	0.98
	Other immunosuppressive drugs		0.76 (0.61-0.87)	0.08	0.97	0.50	0.77
	Cancer		0.92 (0.80-0.98)	1.00	0.91	0.33	1.00
	Diabetes		0.98 (0.89-0.99)	0.86	1.00	1.00	0.98
	Malnutrition		0.94 (0.83-0.99)	0.00	0.98	0.00	0.96
	Other immunosuppressive conditions		0.82 (0.68-0.91)	0.10	1.00	1.00	0.81
	Marginalized		0.96 (0.86-0.99)	0.66 (0.57)	0.93 (0.12)	0.99 (0.02)	0.91 (0.14)
	Health care facility		0.90 (0.78-0.97)	0.38 (0.48)	0.95 (0.08)	0.95 (0.08)	0.97 (0.03)
	**Pulmonary** **acid fast bacilli**						
		Positive	0.92 (0.80-0.98)	0.25	0.98	0.50	0.93
		Negative	0.96 (0.86-0.99)	1.00	0.96	0.67	1.00
	Extrapulmonary (other than lymphadenitis)		0.88 (0.75-0.96)	0.00	0.96	0.00	0.91
	Lymphadenitis		0.94 (0.83-0.99)	N/A	0.94	N/A	N/A
	Disseminated		0.96 (0.86-0.99)	0.00	1.00	N/A	0.96
**Complex features**							
	Active TB disease		1.00 (0.93-1.00)	1.00	1.00	1.00	1.00
	Latent TB infection		0.84 (0.70-0.93)	0.90	0.79	0.76	0.92
	Pulmonary nontuberculous mycobacteria		0.88 (0.75-0.95)	1.00	0.87	0.25	1.00
	**Adverse drug reaction**						
		Gastrointestinal	0.84 (0.70-0.93)	1.00	0.76	0.65	1.00
		Peripheral neuropathy	0.96 (0.86-0.99)	1.00	0.95	0.78	1.00
		Rash	0.90 (0.78-0.97)	1.00	0.89	0.50	1.00
		Other	0.94 (0.83-0.99)	0.00	0.98	0.00	0.96
		Ocular toxicity	0.90 (0.75-0.97)	0.00	0.92	0.00	0.98

^a^Values within parenthesis are standard deviation values.

^b^N/A: not applicable.

^c^TB: tuberculosis.

### Natural Language Processing Performance Adjusted for Adjudication

To understand whether NLP’s relatively low sensitivity and PPV for moderate and complex features might be driven by errors in the manual chart review, rather than errors in NLP, we conducted a post hoc analysis in which all 138 points of discrepancy between the reference standard and index analysis were arbitrated by a clinical expert. The expert found the results to be in favor of NLP in 51.4% (71/138) of cases and chart review in 45.6% (63/138) of cases and found that both were incorrect in 2.8% (4/138) of cases.

After adjusting for the results of adjudication, results for our primary outcome of overall accuracy increased modestly to 98% (95% CI 96.1-98.7) for simple, 96% (95% CI 94.8-97.3) for moderate, and 94% (95% CI 91.3-96.1) for complex features. The sensitivity increased to 78% (SD 25.0) for moderate and 86% (SD 35.0) for complex features, and PPV increased to 93% (SD 14.7) for moderate and 70% (SD 34.2) for complex features (Table 4).

At the feature level (Table 5), adjustment for adjudication resulted in several dramatic improvements, particularly in the area of immunosuppressive drugs and conditions. For example, PPV for both cancer and chemotherapy was only 33% before adjudication but increased to 100% following adjudication. Similarly, for other immunosuppressive drugs, sensitivity was only 8% and PPV was only 50% initially, but it increased to 67% and 100%, respectively, after adjudication.

**Table 4:** Primary and secondary outcomes for natural language processing compared with manual chart review, adjusted for results of adjudication.

Feature complexity	Primary outcome, overall accuracy (95% CI)	Secondary outcomes			
		Sensitivity/recall (SD)	Specificity (SD)	Positive predictive value/precision (SD)	Negative predictive value (SD)
					
Simple	97.8 (96.1-98.7)	96.4 (5.4)	99.8 (0.5)	98.3 (4.5)	99.2 (1.7)
Moderate	96.2 (94.8-97.3)	78.2 (25.0)	93.3 (4.7)	92.7 (14.7)	97.2 (3.2)
Complex	94.1 (91.3-96.1)	86.3 (35.0)	92.8 (8.2)	70.5 (34.2)	98.7 (2.9)

**Table 5:** Primary and secondary outcomes for natural language processing compared with manual chart review, adjusted for results of adjudication at the clinical feature level.

Feature			Primary outcome, overall accuracy (95% CI)		Secondary outcomes^a^							
					Sensitivity/recall (SD)		Specificity (SD)		Positive predictive value/precision (SD)		Negative predictive value (SD)	
**Simple features**												
	Country of birth			0.94 (0.83-0.99)		0.91 (0.28)		0.99 (0.01)		0.98 (0.10)		0.99 (0.01)
	Year of immigration			0.92 (0.80-0.98)		0.92 (0.23)		0.99 (0.02)		0.99 (0.06)		0.99 (0.01)
	Smoking status			0.94 (0.83-0.99)		0.92 (0.08)		0.98 (0.03)		0.85 (0.30)		0.97 (0.02)
	Sputum year			1.00 (0.93-1.00)		1.00		1.00		1.00		1.00
	Pyrazinamide			0.96 (0.86-0.99)		1.00		0.85		0.95		1.00
	Moxifloxacin			1.00 (0.93-1.00)		1.00		1.00		1.00		1.00
	Vitamin B6			0.92 (0.80-0.98)		1.00		0.86		0.84		1.00
	Rifampin			1.00 (0.93-1.00)		1.00		1.00		1.00		1.00
	Ethambutol			1.00 (0.93-1.00)		1.00		1.00		1.00		1.00
	Isoniazid			1.00 (0.93-1.00)		1.00		1.00		1.00		1.00
	Levofloxacin			1.00 (0.93-1.00)		1.00		1.00		1.00		1.00
**Moderate features**												
	HIV status			0.98 (0.89-0.99)		0.95		1.00		1.00		0.97
	TB^b^ contact			0.86 (0.73-0.94)		0.92		0.83		0.67		0.97
	Old TB			0.96 (0.86-0.99)		0.75		1.00		1.00		0.95
	Culture positive			0.88 (0.75-0.95)		0.33		1.00		1.00		0.87
	Polymerase chain reaction positive			1.00 (0.93-1.00)		1.00		1.00		1.00		1.00
	Clinical diagnosis			1.00 (0.93-1.00)		1.00		1.00		1.00		1.00
	Drug sensitivity			0.96 (0.86-0.99)		0.98 (0.03)		0.99 (0.01)		0.80 (0.26)		0.94 (0.10)
	Corticosteroids			1.00 (0.93, 1.00)		1.00		1.00		1.00		1.00
	Chemotherapy			0.98 (0.89-0.99)		0.75		1.00		1.00		0.98
	Other immunosuppressive drugs			0.98 (0.89-0.99)		0.67		1.00		1.00		0.98
	Cancer			1.00 (0.93-1.00)		1.00		1.00		1.00		1.00
	Diabetes			0.98 (0.89-0.99)		0.86		1.00		1.00		0.98
	Malnutrition			0.94 (0.83-0.99)		0.00		0.98		0.00		0.96
	Other immunosuppressive conditions			0.98 (0.89-0.99)		0.5		1.00		1.00		0.98
	Marginalized			0.98 (0.89-0.99)		0.75 (0.50)		0.95 (0.10)		0.99 (0.01)		0.99 (0.01)
	Health care facility			0.92 (0.80-0.97)		0.50 (0.50)		0.86 (0.29)		0.95 (0.06)		0.97 (0.03)
	**Pulmonary acid fast bacillus**											
		Positive		0.92 (0.80-0.98)		0.25		0.98		0.50		0.93
		Negative		0.96 (0.86-0.99)		1.00		0.95		0.67		1.00
	Extrapulmonary (other than lymphadenitis)			0.96 (0.86-0.99)		0.50		1.00		1.00		0.95
	Lymphadenitis			1.00 (0.93-1.00)		1.00		1.00		1.00		1.00
	Disseminated			1.00 (0.93-1.00)		N/A^c^		1.00		N/A		N/A
**Complex features**												
	Active TB disease			1.00 (0.93-1.00)		1.00		1.00		1.00		1.00
	Latent TB infection			0.84 (0.70-0.93)		0.90		0.79		0.76		0.92
	Pulmonary nontuberculous mycobacteria			1.00 (0.93-1.00)		1.00		1.00		1.00		1.00
	**Adverse drug reaction**											
		Gastrointestinal		0.90 (0.78-0.97)		1.00		0.84		0.78		1.00
		Peripheral neuropathy		1.00 (0.93-1.00)		1.00		1.00		1.00		1.00
		Rash		0.90 (0.78-0.97)		1.00		0.89		0.50		1.00
		Other		0.97 (0.89-0.99)		1.00		0.98		0.50		1.00
		Ocular toxicity		0.90 (0.75-0.97)		0.00		0.92		0.00		0.98

^a^Values within parenthesis are standard deviation values.

^b^TB: tuberculosis.

^c^N/A: not applicable.

## Discussion

### Principal Findings

The findings of this study suggest that a commercially available NLP tool can perform very well when compared with the reference standard of manual chart review in extracting useful clinical information from digital text notes in our TB clinic with limited training. This was especially true in the case of straightforward findings, such as prescribed medications, smoking status, country of birth, year of immigration, and sputum conversion date. Unsurprisingly, accuracy decreased slightly as clinical features became more complex, but it remained over 90% for complex features.

One notable finding is that although NLP performed extremely well with respect to specificity and NPV for moderate and complex findings, sensitivity and PPV were considerably lower. These results are in keeping with other studies using free-format clinical notes for complex feature extraction, such as the study by Perlis et al, who reported a sensitivity of 42% and PPV of 78% for the detection of depression [8]. However, these findings are in contrast to the high sensitivity and PPV reported in studies looking at radiology reports, such as the study by Al-Haddad et al, who demonstrated a sensitivity of 97% and PPV of 95% in the detection of intraductal papillary mucinous neoplasms [10]. This discrepancy may be either because of differences in complexity of features or because of differences inherent between radiology reports, which are relatively structured, often with minimal variability from practitioner to practitioner, versus free-format clinical notes, which have less structure and greater variability across practitioners.

In terms of ease of use of a commercially available tool, deploying Pentavere’s DARWEN in our environment was a straightforward installation of their application on a desktop computer. The iterative tuning and relationship modeling for all clinical features took our NLP engineer roughly 4 weeks to complete. The tuning required roughly 6 hours of clinician time to provide clinical context for the NLP engineer, confirm clinical validity of heuristic rules, and perform arbitration of discrepancies between chart review and NLP.

### Strengths and Limitations

Our study is novel in several ways. First, to our knowledge, this is only the third study to explore the validity of NLP for the identification of TB patients and the first to examine dictated consult notes versus radiological reports and structured laboratory results for this purpose [18,19]. Second, research on NLP applications in medicine tend to focus on only a single clinical condition such as the presence of a tumor [10], a diagnosis such as depression [8], or a social condition such as homelessness [9]. In contrast, our study is substantially broader compared with other more commonly published studies, looking at 15 distinct medical and social features. Finally, our study is one of the few to evaluate the performance of a commercially available NLP tool [6,7,12].

Our study has several limitations. First, review of the feature-level analysis reveals that some dichotomous features had very low incidence, making sensitivity and PPV very sensitive to error. Second, our choice to randomly sample for our initial training dataset (n=30) resulted in an undersampling of cases of ocular toxicity because of adverse drug reaction. As a result, the NLP tool was never trained on this feature and subsequently performed poorly for this feature during the final testing set, potentially underestimating the effectiveness of a properly trained tool. This suggests that a real-world application of this technology may require a more purposive sampling strategy than our random sampling approach. Third, our study employed only a single chart abstractor and a single adjudicator. Finally, this study was conducted at a single center, in a focused clinical area, and with a relatively small final test sample (n=49), which may limit the generalizability of our findings. However, the goal of this pilot study was to establish the feasibility of using NLP to extract clinical features from dictated consult notes and to inform the approach to larger future studies.

### Conclusions

NLP technology has been advancing quickly in recent years, and the potential clinical applications are numerous. The findings of this study support the application of extracting clinical features from dictated consult notes in the setting of a TB clinic. Further research is needed to fully establish the validity of NLP for this and other purposes. However, its application to free-format consult notes may be of particular benefit, as it offers a course whereby clinicians can document in their preferred method of narrative free text, with data still available for applications such as research and program quality control initiatives, for example, without the cost and effort of manual chart review.

## Appendix

Multimedia Appendix 1 Corpus sample (synthetic consult notes).

## Abbreviations

AFB: acid fast bacilli
EHR: electronic health record
NER: named entity recognition
NLP: natural language processing
NPV: negative predictive value
PPV: positive predictive value
TB: tuberculosis
