# Supplementary material for: Extracting Clinical Features From Dictated Ambulatory Consult Notes Using a Commercially Available Natural Language Processing Tool: Pilot, Retrospective, Cross-Sectional Validation Study
Source: JMIR Med Inform. 2019 Nov 1;7(4):e12575. doi: 10.2196/12575 (PMC6913750; doi:10.2196/12575)
Supplement: Multimedia Appendix 1 [file medinform_v7i4e12575_app1.pdf]

TB Clinic Sample Note – First Assessment

Dear Dr Doctor

Mr I. Person was seen in the Tuberculosis clinic Oct 20, 2016, with the aid of an interpreter. He is a 75 year old Tagalog speaking man who presents with symptoms and imaging suggestive of active pulmonary TB.

Mr Person has had a cough “lifelong”. Over the past 6 to 7 months it is increasing in frequency and severity, and is now accompanied by shortness of breath. It is non-productive; there is no fever, chest pain, hemoptysis, nights sweats or changes in his weight. His appetite remains intact, but his energy level is diminished. He typically can walk an 18 hole golf course to play a round; this summer he had to take a golf cart due to shortness of breath and disabling fatigue.

He presented to you approx. 1 month ago. A CXR was reported as showing a left upper lobe infiltrate. A course of moxifloxacin was administered with slight symptomatic improvement. A repeat CXR revealed non resolution of the inflltrate and the reporting radiologist included the possibility of TB in the differential. He presents today for urgent assessment.

Mr Person was born in the Phillipines, and immigrated to Canada in 1999. He has a past history of previous TB approximately 40 years ago, which was treated in the Phillipines for a number of months. He is unable to recall the names of any of the drugs or the specific duration of therapy. He is otherwise well and denies any chronic illness, or maintenance medications, although prior to his visit to yourself in the walk in clinic, he had not sought medical attention since arrival in Canada. He has no known drug allergies.

He is currently retired, but previously worked as a prison guard in the Phillipines. He has a 100 packyear smoking history, but quit approx. 4 years ago. He does not consume alcohol, does not use illicit drugs. He is married with 3 grown children, and lives in a seniors complex with his wife. He is unaware of his HIV status but denies a lifestyle that would put him at risk for acquisition of infection.

His family history is significant for autoimmine disease (mother rheumatoid arthritis, sister SLE).

On examination he appears thin, but in no distress. Vitals revealed a HR 85 and regular, BP 130 /60 it the right arm sitting. Head and neck exam revealed a clear oropharynx and no cervical or supraclavicular lymphadenopathy. Chest was clear to auscultation. Heart sounds were normal with no murmurs. Abdominal exam was unremarkable with no organomegaly. There is no axillary adenopathy.

CXR obtained today revealed a left upper lobe nodular infiltrate with a trace effusion. There is a density in the medial aspect of the infiltrate that is suspicious for an actual mass. The right lung appears clear and the mediastinum is not bulky.

In summary Mr Person is a 75 year old Filipino born man, previous smoker with a history of previous TB who presents with a worsening cough, fatigue and a infiltrate and possible left upper lobe mass, non responsive to a course of antibiotics. Given his history it is possible we are dealing with active pulmonary

TB, but I am also concerned re an actual left upper lobe mass and in the context of his smoking history, malignancy is in the differential. Hence today we will perform an induced sputum for AFB smear and MTB culture. We will arrange for urgent CT thorax, with contrast, to better visualize the LUL possible mass. He has been instructed to remain in respiratory isolation at home, and to wear masks, pending the results of his AFB smear. We will see him back in followup in 1 week once we have the results from his concentrated smear and CT thorax. At that time, if the CT is suspicious for malignancy we will proceed with bronchoscopy.

Thankyou for involving us in the care of this pleasant gentleman. We will see him back here in 7 days.

TB Clinic Sample Note – Follow-up #1

Dear Dr Doctor

Ms Young Lady was seen in followup in the TB clinic on Oct 24 2016 for her latent TB infection.

As you know we first saw Young in August 2016, as she is the household contact of an individual with active pulmonary TB. She is TST positive, but showed no evidence of active TB on history, physical exam or on chest imaging, and sputum culture was MTB negative. Treatment of her LTBI was initiated with INH 300mg daily, and Vit B6 25 mg daily on August 26. She presents today for her 2 month followup

She has been compliant with her medication, is not consuming alcohol, and has remained in her usual state of health. Her only other medication currently includes Alesse. She has no symptoms compatible with INH induced hepatotoxicity or neurotoxicity. Her last LFTs were within normal limits.

In summary Ms Lady is undergoing treatment of LTBI with INH and Vit B6. She is tolerating treatment well. I have provided her with a prescription for Isonizid 300mg and VitB6 25mg daily and we will see her back in 1 month. We will draw LFTS today. Should her enzyme levels be of concern, we will contact her by phone with instructions on how to manage her medication.

Thank you for involving us in the care of this pleasant young woman.

TB Clinic Sample Note – Follow-up #2

Dear Dr Doctor

Miss Happy was seen in follow-up on Oct 24th 2016 for her disseminated TB.

As you are aware a diagnosis of fully sensitive disseminated TB was made in July 2016. Treatment was initiated with 4 first line drugs, and Ethambutol was discontinued in early August once sensitivities were known. She completed her 2 month intensive phase Sept 15 2016, pyrazinamide was discontinued and she was stepped down to INH, Rifampin and Vit B6 for the remainder of her continuation phase. Sputum from August 15th was smear negative, culture positive. Cultures from sputum induced Sept 8th 2016 remain negative to date (now 6 weeks out). We plan to treat her for a minimum of 9 months, and may possibly extend treatment to 1 year, given the burden of disease.

She was released from respiratory isolation approximately 1 month ago, and has returned to work. She is very pleased to be back into a normal routine.

Today she feels well. She is delighted with her state of health. Her energy is returning and she is gaining weight (current weight is 56 kg, up 2 kg since her last visit). Her review of symptoms is negative. The parsethesias she was previously experiencing have resolved with the increase in her Vit B6 to 50mg daily.

She remains on DOT and is tolerating her medication well. Transaminases have been slightly elevated (approx. 3x normal) since initiation of treatment, but she remains asymptomatic from a GI standpoint. Bloodwork for LFTs and enzymes, and CBC from today is still pending. She remains on her current antihypertensives (HCT and Amlodipine). She is abstaining from alcohol.

I have provided her with a prescription for INH 300 mg, Rifampin 600mg and VitB6 50mg daily and we will see her back in followup in 1 months time. Of course if her liver enzymes continue to rise, we will need to change her current therapy. Provided she continues to improve we plan to repeat her CT abdomen and CXR at 8 months, to assess resolution of her psoas abscessus and pulmonary infiltrates. The response will help to guide her treatment duration.

Thankyou for allowing us to participate in her care. We will see her in a month.
